# Designing and evaluating tasks to measure individual differences in experimental psychology: a tutorial

**DOI:** 10.1186/s41235-024-00540-2

**Published:** 2024-02-27

**Authors:** Marc Brysbaert

**Affiliations:** Department of Experimental Psychology, Ghent University, 9000 Ghent, Belgium

**Keywords:** Tutorial, Individual differences, Statistical analysis

## Abstract

Experimental psychology is witnessing an increase in research on individual differences, which requires the development of new tasks that can reliably assess variations among participants. To do this, cognitive researchers need statistical methods that many researchers have not learned during their training. The lack of expertise can pose challenges not only in designing good, new tasks but also in evaluating tasks developed by others. To bridge the gap, this article provides an overview of test psychology applied to performance tasks, covering fundamental concepts such as standardization, reliability, norming and validity. It provides practical guidelines for developing and evaluating experimental tasks, as well as for combining tasks to better understand individual differences. To further address common misconceptions, the article lists 11 prevailing myths. The purpose of this guide is to provide experimental psychologists with the knowledge and tools needed to conduct rigorous and insightful studies of individual differences.

## Introduction

Scientific research uses two methodologies to establish relationships between variables: the experimental and the correlational method. In experimental research, a variable is intentionally manipulated to observe its effect on another variable while controlling for extraneous factors. Correlational research examines the association between existing variables without the ability to manipulate them.

Cognitive psychology emerged from the experimental tradition (Cronbach, 1957; Neisser, 1967) and long defended the experimental method as the only acceptable method, because only experimental manipulations could demonstrate causal relationships between variables (Winston, 1988). The preferred statistical tests were t-tests and analysis of variance.

What cognitive psychologists did not mention was that their independent variables were often not true experimental variables that could be freely manipulated. A true experimental design requires that instances can be randomly distributed across conditions (or ideally studied in all conditions of interest). This is the case, for example, in a Stroop experiment,^1^ where participants take part in both the congruent and incongruent condition and the stimulus words can be assigned to the congruent and incongruent condition at will.

Many variables studied by cognitive psychologists cannot be randomized, however, because they are inherent properties of people (or stimuli). For example, Woodhead and Baddeley (1981) compared a group of people who were good at memorizing faces with a group of people who were poor at it. They found that the good group was also better at remembering paintings but not at remembering words, suggesting a dissociation between the retention of visuospatial and verbal material. Importantly, Woodhead and Baddeley (1981) were not able to randomly place people in the condition of good and bad face recognition. All they could do was select people based on an existing difference, making their design a correlational design even though the data were analyzed with analysis of variance. The findings of the study are best summarized by saying that there was a correlation between memory for faces and paintings, but not between memory for faces and words.

Daneman and Carpenter (1980) understood the correlational nature of participant differences in memory better. So, when they investigated whether people with high working memory capacity would understand texts better than people with low memory capacity, they did not “manipulate” working memory capacity, but looked at the correlation between working memory capacity and reading comprehension. The correlational approach gained further impetus, when it was discovered that individual differences in working memory correlated strongly with intelligence (Engle et al., 1999). Short-term memory tasks have been part of intelligence tests since the first test was proposed by Binet and Simon (1908), building on the work of Jacobs (1887), but working memory scores correlated even more with intelligence tests, and several tasks were developed to best measure working memory capacity. The quality of the tasks was assessed by correlating them with existing working memory tasks and performance on intelligence tests (Conway et al., 2005; Kane et al., 2004).

Closely related to working memory are executive functions, the functions needed to initiate and perform tasks, while ignoring distractors. Here too, consistent individual differences have been observed and linked to theories about the nature of the functions (Friedman & Miyake, 2017; Miyake et al, 2000), and again research has been devoted to finding tasks that optimally measured the various functions (Karr et al., 2018; Rey-Mermet et al., 2018).

Individual differences are also important for cognitive psychologists seeking to translate their findings into practical applications. Chan et al. (2021) pointed out the relevance of executive functions to effective leadership and management and emphasized the role of efficient attention management in facilitating rapid adaptation to new and dynamic task demands. Translating theoretical insights into concrete strategies, however, requires the development of tasks that can reliably assess individual differences in executive functions and establish their correlation with job performance. Only by bridging the gap between research and practice can the full potential of theoretical knowledge be harnessed to optimize applied outcomes. Experimental tasks offer a distinct advantage in this regard because they provide performance-based measurements that may provide a more accurate assessment of actual performance than subjective self-assessments (Rothlind et al., 2017; Zell & Krizan, 2014).

The similarity between research in which groups of participants are selected and research that correlates task performance with existing individual differences became more apparent when regression analysis allowed the inclusion of categorical variables in addition to continuous variables. This showed that there was no difference between analyses with categorical variables (t-test, analysis of variance) and regression analysis with continuous variables. It also became clear that categorizing continuous variables in factorial designs (a low versus high group) was poor for the power of the design and hindered understanding of the underlying processes (Balota et al., 2012; Royston et al., 2006).

Because of the above evolutions, cognitive researchers increasingly test theories by studying existing differences between participants (e.g., Goodhew & Edwards, 2019; Unsworth, 2019). For this research, they need statistical methods that were not taught to them and about which little information can be found in the cognitive literature. This is a problem not only when they set up a study, but also when they are asked to evaluate such studies (as an examiner, reviewer, or editor). This article is intended as a gentle introduction to the literature of studying individual differences. We begin with the basics of test psychology.

## The basics of individual differences testing

### Standardization

Developing a robust and reliable task for assessing individual differences requires a significant investment of time and effort, which is underestimated by experimental psychologists, who often rely on self-selected stimuli that lack typical test qualities. The latter is illustrated by the previously introduced memory study of Woodhead and Baddeley (1981). The authors described the stimuli they used as follows (p. 369): “Each of the three tests comprised slides of 100 items, of which 50 were targets and 50 were distractors. The stimuli in the faces test consisted of black-and-white photographs of the faces, including neck area, of unfamiliar actors whose names were unlikely to be known to the public. The paintings test consisted of representational nineteenth and twentieth century paintings, mainly scenes and objects; a few contained human figures, but these were not conventional portraits. The words test was composed of commonly used three-, four-, and five-letter nouns, verbs, and adjectives.”

Such untested, ad-hoc stimuli may suffice for comparing extreme groups but often fall short in studies of individual differences. This is because the instruments lack the sensitivity to capture variation across the entire range of performance, there is no assurance that the stimuli accurately assess a single skill, and there is no guarantee that performance differences between participants remain consistent across repeated assessments. Furthermore, the authors' assertion that their stimuli measure long-term memory is solely based on the theoretical framework they employ, with no independent validation evidence to support the claim.

Assessing individual differences necessitates the use of validated tasks or protocols that are delivered in a standardized manner. Experience has shown me that developing such tasks can easily take more than a year. This significant time commitment is primarily due to the rigorous evaluation and refinement of the task through multiple iterations (as described below), to ensure that it effectively captures meaningful individual differences. This level of effort has two implications: (1) if you do not have the resources to invest in creating a proper new task, it is more advisable to utilize an existing standardized test, and (2) if you have successfully developed a well-validated task, it is crucial to make it publicly available so that others can build on your work.

### Reliability

Because the research design is a correlational design, it is important that the test scores be stable, a requirement called reliability. You cannot interpret a correlation between two variables if you do not have information about the reliability of the variables. This is especially true if you find a low correlation, because a low correlation between two variables can have two origins: the variables are not related at the population level, or the variables were not measured reliably. A variable cannot correlate with another variable any more than it correlates with itself.

Suppose you have created a test of a stable personality trait (working memory capacity, executive functions, vocabulary size, …) and you ask your participants to take the test twice, one week apart. If you find a correlation of *r* = 0.1 between the two scores, it is meaningless to correlate one of the scores with performance on another test because the scores do not reflect a stable trait of your participants (you get completely different scores the second time than the first time).

The stability of test scores over subsequent testing is called ***test–retest reliability***. Someone who scores high on the first testing is expected to score high on the second testing; someone who scores low the first time is expected to score low the second time, at least if the trait is assumed to be a stable trait (an exception can be made for features that vary greatly in time, such as context-driven emotions).

Because it is not always feasible (or desirable) to obtain test–retest scores with some time in-between, another way to measure the reliability of test scores is to look at ***internal consistency***. If a test consists of two or more items, you can correlate performance on the items. There are different techniques (see below), but the general idea is very simple. If a test measures a single trait, the expectation is that someone who scores low on one item will also score low on the other items; someone who scores high on the item will also be expected to score high on the other items. Internal consistency cannot always be assessed, such as in timed tasks where participants must complete as many items as possible within a given time frame.

Internal consistency and test–retest reliability offer different but complementary insights into the quality of a measurement instrument (McCrae et al., 2011; Revelle & Condon, 2019). Internal consistency reflects the coherence of items, while test–retest reliability assesses the stability of test scores over time. Therefore, obtaining measures of both internal consistency and test–retest reliability is advantageous when feasible.

### Norms

When the same test is administered to different groups, we have additional information about the relative performance of the groups. In other words, there are norms. This has the advantage that scores can be compared between studies. If researcher A gives their own test and finds a mean performance of 65% (SD = 10) and researcher B gives another idiosyncratic test with a mean performance of 80% (SD = 5), we cannot compare performance of the two groups. But if both groups of participants were tested on the same test, we immediately see that Researcher B's participant group scored higher than Researcher A's participant group, and that the variability of Researcher B's participants was smaller than that of Researcher A. Thus, Researcher B worked with a more selected group of participants than Researcher A. This is likely to affect the pattern of correlations that will be found between test scores and other variables, as we will see below.

Normed tests and tasks also help to correctly evaluate standardized effect sizes commonly used in meta-analyses. Suppose a researcher in a study finds that a group of 100 female students scored higher on average on an IQ test (*M* = 115, SD = 3.0) than a group of 100 male students (*M* = 113, SD = 3.3, *t*(198)−  = 4.48, *p* < 0.001, two-sided). Translated to a standardized Cohen's d effect size, this would be a difference of *d* = 0.63, which seems like a large effect size for a difference of only 2 IQ points. The reason for the high d value is the small standard deviations in both groups (indicating that the groups were strongly selected on IQ). If the standard deviation from the norming study in the full population is used (SD = 15), the estimated effect size becomes a more realistic *d* = 0.13.

If necessary, norm-referenced tests also offer a systematic and principled approach to identify outliers, enabling researchers to restrict their study to participants within the mainstream population (e.g., individuals with normal reading abilities).

### Validity

Finally, it is not enough that a test is administered in a standardized manner, is reliable and has norms to be a good test. For example, we could design a standardized test that requires participants to copy a printed text by hand, and we could design a scoring mechanism that is reliable and gives us norms for different groups of people. Still, we would (hopefully!) hesitate to use this test as an indicator of intelligence, working memory capacity, or cognitive control. We would hesitate because we would doubt whether the test measures what it is supposed to measure, a test characteristic called validity.

The validity requirements for tests were gradually tightened in the twentieth century. In the beginning, it was sufficient that the content of the test seemed applicable. For example, we would not accept copying texts as a test of verbal skill, but we could accept a dictation task as a valid test because people must know the words in order to write them correctly (certainly in English). This validity criterion is called ***content validity***. It is the validity criterion that cognitive researchers such as Woodhead and Baddeley (1981) rely on when selecting stimuli for their experiments.

Content validity is not enough, however, because often we do not know which content best measures the trait we are interested in. To know that, we additionally need to find out if the test correlates with a real-life consequence of what we hope to measure. If we think working memory ability is related to intelligence, we want to see a reasonably high positive correlation between scores on a working memory task and scores on an intelligence test or other indications of intelligent achievement (e.g., school performance). This is called ***criterion validity***. At the very least, we expect our new test to correlate well with an established test that is supposed to measure the same trait. This is called ***convergent validity***. We also expect no correlations with tasks that are assumed to measure other, independent traits (e.g., how friendly the participant is when taking the test), a requirement called ***discriminant validity***.

Although content validity and criterion validity cover most of the concerns we can have about the usefulness of a test, in the mid-twentieth century it became clear that they are not sufficient. The question that remained is how certain can we be that the traits we think we measure really exist. What evidence do we have for human characteristics such as working memory capacity or intelligence, beyond the fact that people differ in performance on some tests we devised?

Hanson (1993) pointed to the possibility that intelligence as understood in the Western world could be the result of the specific tasks chosen by Binet and Simon (1908). He wondered how the world would have differed if the following tasks had been included in the intelligence test:

1. A name recall scale: how well can you remember the names of people you have just been introduced to?
2. A math scale: how good are you at arithmetic and algebra?
3. A first impression scale: how good is the first impression you make?
4. An exposition of ideas scale: how convincing is a text you write on a topic you had 5 min to study?
5. A small-talk scale: how well can you have an interesting conversation with someone you've never met before?
6. A bullshitting scale: how well do you perform in a discussion on a topic you know nothing about?
7. A follow-the-directions scale: how well can you follow a six-step procedure explained once?
8. An adult sports scale: How good are you at playing golf and tennis?
9. An SES scale: what is your parents' socioeconomic status?

Hanson (1993) mused on how education would have adapted to the alternative view of intelligence. Indeed, according to some sociologists, education has been designed by powerful groups in society not to teach young people the skills they need at work, but to limit access to coveted positions (Dore, 1997).

To be sure that a test is measuring something of interest, we need ***construct validity*** (Cronbach & Meehl, 1955): A test must match the proposed interpretation, and that is only possible if you have a correct picture (theory) of the complete situation. Unfortunately, it is next to impossible to meet this requirement. Cronbach (1989, p. 151) characterized it as “a lengthy, even endless process”.

The need for construct validity is a real stumbling block for researchers who want to develop a test, because they usually do not have the required overarching theory. It is also an easy argument for reviewers and editors to reject a manuscript they are not interested in, because it is a criterion that can easily be stretched to the point of being unattainable.

Fortunately, scholars have since come up with more pragmatic criteria for test validity. The following is based on ideas by Kane (1992, 2013) and applied to language tests by Chapelle et al. (2010).

### Kane’s interpretation/use argument (IUA) of validity

According to Kane (1992, 2013), a test's validity requirements depend on the use researchers envision for their test or task. This requires researchers to be explicit about the use of their test and argue why the proposed test is valid for its intended purposes. Tests with high stakes and real-life consequences (e.g., access to a particular type of education) require more stringent criteria than a low-stakes test used for psychological research.

At the lowest level, the ***observation level***, a researcher wants to develop a good task to measure a known feature. For example, a researcher wants to create a new vocabulary test or to translate an existing test for research purposes. Then, in principle, it may be sufficient to ensure that there are no content validity problems and that the test as a whole has good reliability (e.g., is not too easy or difficult for the intended group, has enough items of adequate difficulty, and is scored appropriately). In addition, assessment of criterion validity is appreciated, but is not expected to be problematic given what is already known about the test (in other languages).

The next level is the ***target level***, where an interpretation of the scores is made. For example, the vocabulary test is no longer used as an estimate of vocabulary size, but as an indication of language ability. Or an N-back task is no longer used to make statements about the N-back task, but as an indication of working memory capacity (Jaeggi et al., 2010). At this level, generalization claims are made that should be supported by evidence. The least one wants to see is convergent and discriminant validity. For example, if a new test of emotional understanding is presented, one would want to see evidence that the new test correlates with existing tests of emotional understanding and that the test measures something different from a regular intelligence test.

**Fig. 1:**
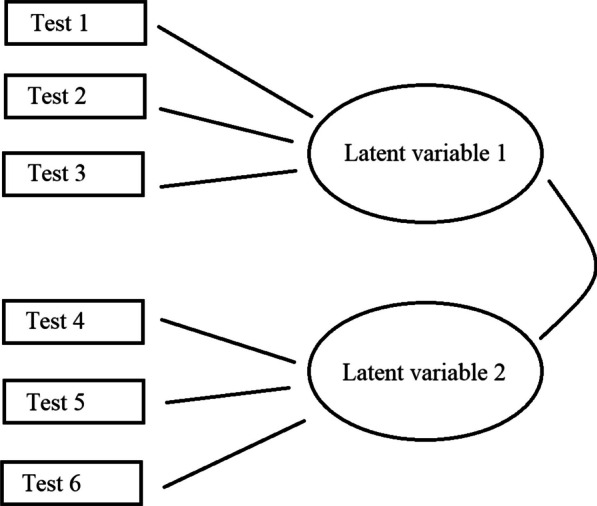
Test scores are used to estimate the most likely scores on latent variables (constructs). In this example, three tests are used to measure latent variable 1 (e.g., emotion perception) and three more test scores are used to measure latent variable 2 (e.g., analytical intelligence). On the basis of the correlations between the tests, the most likely loadings of the tests on the latent variables are calculated, together with the most likely correlation between the latent variables. The latent variables are traits that cannot be observed directly, because every test is a combination of variance due to the latent variable and methodological variance

At the third level, the ***construct level***, the researcher wants to make statements that go beyond the trait itself, for example, about the importance of the trait for performance on tasks that measure another trait. This requires a theory of why the traits are needed and how they are related. At this level, the traits are represented by so-called latent variables or constructs (Fig. 1). These are variables that can no longer be directly observed, but are linked to observable characteristics via theory. For example, it can be argued that there is no pure test of "working memory capacity," because each test is also influenced by methodological choices and possibly by other latent variables. However, if different tests partially measure the same latent variable (the construct), they will correlate with each other and these correlations can be used to obtain a good estimate of the latent variable. So, Hanson's (1993) concern that IQ scores depend on the given subtests can be examined by looking at the latent variable of general intelligence as measured by one set of subtests and the latent variable as measured by a largely different set of subtests. If both sets of subtests measure the same construct (general intelligence), then the scores on the latent variables should be highly correlated, as found by Johnson et al. (2008). Otherwise, Hanson's criticism applies and there is no such thing as general intelligence.

At the fourth level, the ***real-life level***, tests are used to predict performance outside the research domain. This is where the test begins to have societal impact. Again, researchers must make good arguments for such use. These will be based in part on the theory developed at the construct level, but also on the correlation between the test result and the actual consequence. Is the correlation high enough to have practical implications? For example, can a vocabulary test be used to estimate the language proficiency of students who want to attend a university where a language other than their dominant language is used? If so, why; if not, what are better alternatives and why?

Finally, at the fifth level, the ***decision level***, test scores are used to grant or deny privileges that people want, or to impose conditions that people try to avoid. At many universities, for example, students speaking another language must achieve a certain score on a language proficiency test before they are admitted. Here you can clearly see the importance of construct validity as discussed by Cronbach and Meehl (1955). How confident can we be that the test measures what it purports to measure and that the measured trait has the implications assigned to it (e.g., that inadequate mastery of the language will prevent students from learning the content needed to earn a degree). Otherwise, the test misses its purpose and is only used to deny a desirable situation to people who are not powerful enough to question the value of the test. In addition, we would like to see the arguments for the decision criterion used. Why are scores below this level inadequate and scores above that level good enough?

Importantly, according to Kane (1992, 2013), the validity criteria required at the fourth and fifth levels are much more demanding than those at the first three levels, where validity refers only to usability within the research framework. As long as experimental research is limited to basic research (trying to understand cognitive processes), researchers remain largely within the first three levels. This is different when basic findings are translated into practical applications (e.g., selection tests). Then, the fourth and fifth levels become as important for experimental psychologists as for other psychologists working on applied issues with social impact.

In the following sections, good practices are described for three common situations roughly coinciding with Kane's first three levels.

### Developing a new test/task

A first situation arises when we see a need for a new test. We want to investigate something for which no test yet exists, or we think we can make a better test for an established ability. Then the following considerations should be considered.

### Why is a new test needed?

To convince readers of the need for a new test or task, it is important to articulate its unique value to the field. If the new test measures a new skill, explain the importance of assessing this particular skill and how it expands our understanding of cognition and behavior. If the test evaluates an existing skill, highlight the shortcomings of existing measurements and how the new test addresses these limitations. By highlighting the distinctive features and improvements of the proposed test, you can convince readers to accept and start using your test. Otherwise, they will stick to established, widely accepted tests because these are less criticized.

At the same time, experience shows that reviewers and editors are often overzealous in judging the need for a new test, fearing that "something similar already exists." Even when there is a considerable overlap with existing tests, a methodologically strong, new test is almost always interesting because it increases the precision with which a latent variable can be measured (Fig. 1). If we accept the distinction between tests (manifest variables) and skills (latent variables or constructs), then no test measures the skill completely and no two tests are exactly the same. The strength of the approach lies in the convergence of multiple test results.

Campbell and Fiske (1959) introduced this approach and called it the multitrait multimethod matrix. It emphasizes that no single test fully captures a latent variable and that each test is likely to be influenced by multiple latent variables. Therefore, it is unrealistic to expect a one-to-one correspondence between tests and skills. Within this framework, there is no compelling reason not to publish a well-designed test, even if it measures the same construct as existing tests, because it is likely to differ in the type of stimuli presented and answers asked, making it a valuable addition.^2^

The usefulness of the multitrait multimethod approach can be seen in research on individual differences in face perception (Bobak et al., 2019; Bruce et al., 2018; Rossion, 2014; Young & Burton, 2018; Zhou & Jenkins, 2020). For this field of research, it would be a great loss if they had only one face recognition test to work with, because much information is gained by comparing performance on different tests (Esins et al., 2016; Stantic et al., 2022; White et al., 2022). On which aspects do they converge? On what aspects do they diverge, and what can be learned from this? Similarly, when examining the correlation between face perception and analytical intelligence, it is better to have several tests of analytical intelligence, rather than one, so that the latent variable of analytical intelligence can be properly estimated.

Still, it is essential for test developers to thoroughly justify the need for a new test and demonstrate the soundness of their proposed measure. The scientific community does not welcome a proliferation of poorly conceived tests that generate more confusion than clarity. To effectively persuade readers, test developers must clearly articulate the rationale behind their test creation and provide compelling evidence supporting its validity and utility. As we will see in the section devoted to the evaluation of tests, two critical pitfalls that must be avoided are situations in which a new test assesses a known skill in an unintended way that differs from existing measurements (jingle fallacy) and circumstances in which a new test fails to capture the intended skill but instead measures a related skill for which there are already good tests (jangle fallacy).

### Content validity

A test is more likely to be adopted if the content matches users' expectations. Developers need very strong theoretical and empirical evidence to convince users that a test with unexpected content is useful (has validity). Thus, for a vocabulary test, users expect existing words that vary in difficulty. For a test of emotion perception, users expect to be presented with emotionally charged stimuli to which participants must respond.

Content can be based on developers' intuitions or previous research. Mortillaro and Schlegel (2023) point out that building content on an existing theory is a particularly good idea. They discussed the development of tests to measure emotion understanding. Several theories have been proposed about the processes involved, or classifications about the types of emotions worth distinguishing. Building the content of a test on one of these theories provides a firmer foundation than starting from scratch, and has the added benefit of assessing the quality of the theory or classification.

### Make sure you have good estimates of correlation coefficients

Test quality is based on correlations. So, it is important you get accurate estimates of the correlation coefficients. This requires that your participants are motivated and cooperative and that you have enough observations.

**Fig. 2:**
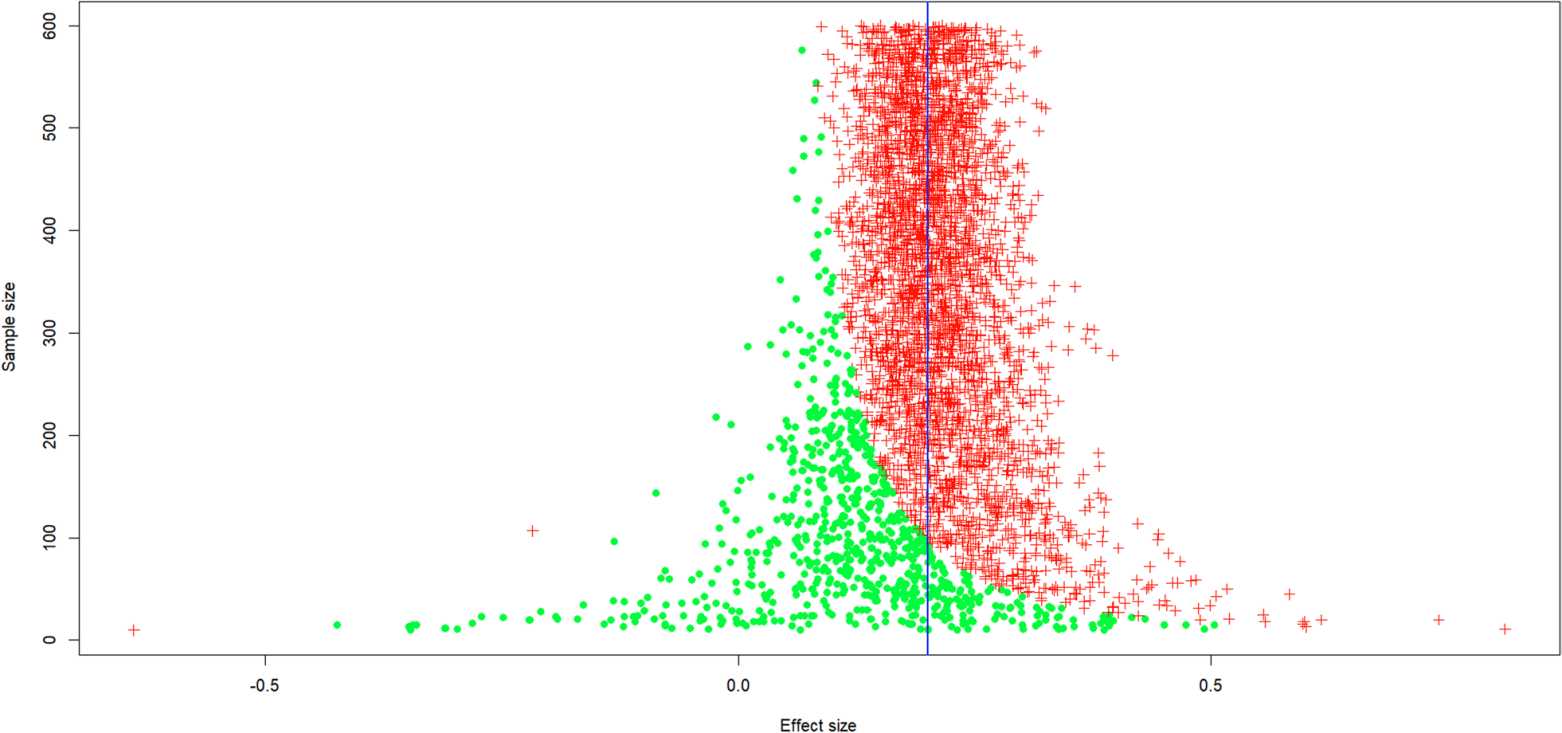
A simulation of 4000 correlation studies with sample sizes from *N* = 10 to *N* = 600. Expected value is *r* = .2. The plot shows that correlations go from − .60 to + .70 for small sample sizes, whereas all correlations are between .1 and .3 for large sample sizes. Each red + is a significant correlation (*p* < .05), each green o is a non-significant correlation. Sample sizes of *N* = 200 are the minimum needed to get stable correlation estimates; sizes of *N* > 400 are better

As for the number of participants to be tested, the minimum is 200 participants. Figure 2 shows the range of correlations you can expect to find when the true correlation is 0.2 as a function of sample size *N*. Sizes below 100 are too dangerous because you risk finding a negative correlation instead of 0.2. When *N* is 200, you will nearly always obtain a positive correlation and the correlations are likely to fall between *r* = 0.0 and *r* = 0.4. Still better is *N* > 400, because then the obtained correlation is likely to fall between *r* = 0.1 and *r* = 0.3, in line with the true correlation (see also Schönbrodt & Perugini, 2013).

Large sample sizes are feasible now that studies can be conducted online and participants no longer need to come to a special lab room. The main caveat to online testing is that procedures must be installed to remove data from inattentive participants in a principled manner. The best way to avoid data loss is to make the experience agreeable for participants so that they feel valued (for example, by being informed of requirements, their progress and level of performance). Because of past exploitation (participants paid almost nothing for demanding tasks), it seems that some online platforms are better avoided for test evaluation because participants are no longer motivated to answer honestly or because answers are provided by bots (Eyal et al., 2022; Hays et al., 2015; Moss et al., 2023; Muraki et al., 2023). Additional data cleaning procedures may involve examining correlations between participants (particularly effective for performance tests where items differ in difficulty), assessing response times, and identifying recurring patterns of responding that disregard the actual content being presented. Pioneering algorithms for identifying careless responses are emerging (Ulitzsch et al., 2024; Wind & Wang, 2023), and more are expected to follow in the near future. What must be avoided, is that the selection of participants depends on what seems to “improve” the quality of the test (Crede & Harms, 2019; Flake & Fried, 2020).

### Reliability

We start with the assumption that your test measures a single trait. You plan to use the sum score of all items as the best estimate of a participant's performance. The first thing you then want to do, is look at the reliability of your test. Reliability below 0.7 is not good (unless you combine tests, as we will see later). Reliability above 0.8 is better. There are many ways to calculate reliability. If you only have one test session, you cannot calculate test–retest reliability, but you can calculate internal consistency. The best known index for this is Cronbach's alpha, because it has been in statistical packages for a long time. Recently, alpha has become less popular because it makes assumptions that are rarely met in psychological studies (Revelle & Condon, 2019). The recommendation is to perform McDonald's omega (or omega total), although the differences between the two are usually small (with omega being the larger). The coefficients can be obtained using the psych() library in R (Revelle, 2023) with the following commands.^3^

library(psych).

alpha(mydata) # look for the value of alpha.

omega(mydata) # look for the value of omega total.

The output of the alpha() command also gives you the correlation between an item and the rest of the items (you'll find it under the r.drop column). This can be used to remove bad items. Good items are items with high positive item-rest correlations, because this means that someone who scored low on the item also scored low on the rest of the items, and someone who scored high on the item also scored high on the other items. If the dependent variable is Likert ratings, you can often drop items with item-rest correlations lower than *r* = 0.3 or even *r* = 0.4. If the dependent variable is based on performance (accuracy, response time), correlations are lower and you should often keep items with *r* > 0.2. The lower the item-rest correlations, the more items must be included in the test to achieve good test reliability.

Experimental psychologist have ignored the reliability issue for a long time (and many still do). Just to give one example, results of multiple regression analyses are happily reported without any assessment of variable reliability. Still, it remains true that a variable with low reliability cannot correlate much with other variables.

Recently, awareness has dawned that many measures of individual differences in experimental psychology are far from reliable (Dang et al., 2020; Elliott et al., 2020; Hedge et al., 2018; Heyman et al., 2018; Noble et al., 2021; Rouder & Haaf, 2019; Siegelman et al., 2017; Staub, 2021). Two factors are involved. First, experimental psychologists work with variables that are much noisier than verbal responses (reaction times, electrical activity, brain responses). Second, most effects in experimental psychology involve a difference between two conditions (e.g., Stroop effect, interference effects, …). The reliability of difference scores is known to be low when both conditions are highly correlated (Allison, 1990; Cronbach & Furby, 1970), as is almost always the case in reaction time experiments. Some headway in tackling these issues is being made (e.g., Snijder et al., 2023), but progress has been slow and probably will require the development of new, better tasks (Rouder et al., 2023).

It is important to note that reliability is sample dependent. A test with good reliability in one sample may not have the same reliability in another sample. This will be the case when the new sample scores higher or lower than the original sample (giving rise to ceiling and floor effects), when the variability in the new sample is smaller than in the old sample (range restriction), or when the participants cannot be compared (e.g., different groups of people). Therefore, it is important for test developers to be transparent about whom they have tested and for whom the test can be used. It is also necessary to have a representative sample of the target population so that the test user can expect similar reliability if they use the test in the same population. It is also important for test users to always report the reliability of the findings obtained in their study.

### Factor structure

Another aspect you may want to check is how well your test measures a single factor (latent variable) by running an exploratory factor analysis.^4^ This can be checked in R with the command.

fa.parallel(mydata).

**Fig. 3:**
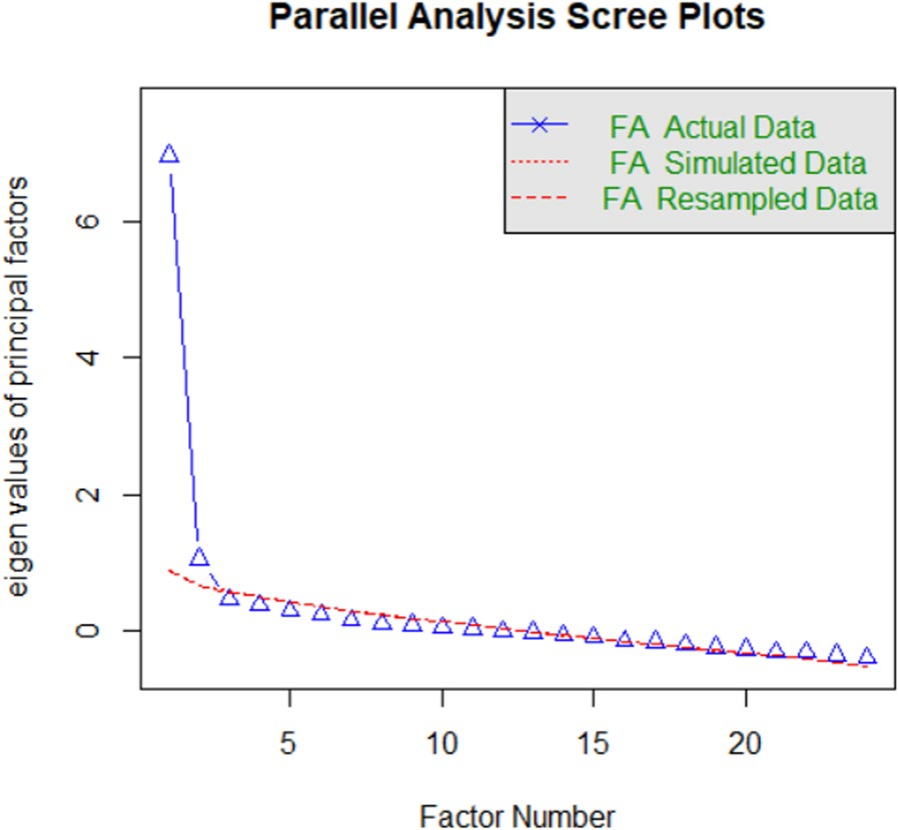
A scree plot of a test with 24 items. The ordinate shows the weight (eigenvalue) of each factor. A test measuring one factor is characterized by a steep drop from factor 1 to factor 2. Ideally, the weight of the observed data in factor 2 falls below random simulated or resampled data. Then there is no doubt that the test measures a single factor. Most of the time, you will find that a few factors remain above the random data. This is not worrying as long as the difference is small and the elbow of the curve is clearly between factor 1 and factor 2

The command will return a scree plot as shown in Fig. 3 for a test with 24 items. A factor analysis tries to summarize the correlations between items with a smaller number of factors. If the correlations between items are all positive and of similar (high enough) magnitude, it is possible to describe the correlations quite well by assuming that they all measure a single factor (as was assumed for the tests in Fig. 1). If, however, some items correlate more with each other than other items, a second factor is needed to describe the distribution of correlations, and sometimes a third, a fourth, and so on. In the end, the correlations are perfectly described when the number of factors is the same as the number of items. At that time, however, the factors no longer account for any variance of interest.

If a test measures a single factor, we expect a large drop in the weight of the factors (called eigenvalues) from factor 1 to factor 2 (as shown in Fig. 3). Ideally, the weight of factor 2 falls below what is obtained by randomly shuffling the data or simulating the design with random data. Then the evidence is clearly in favor of a unifactorial model. Usually, however, the weights of factor 2 (and perhaps a few more) will be slightly above the random data, suggesting that the factor also has a real influence. This is usually not a major problem, as methodological issues (such as a group of items with similar performance or reverse-coded items) can easily account for the extra factor(s). Trying to eliminate these minor irregularities can do more harm than good, especially if subsequent analyses show good test–retest reliability and good correlations with other tests (see below). The reason for the potential harm is that trying to eliminate slightly anomalous items may reduce the heterogeneity of the items, so that the test covers only a fraction of what the test is supposed to measure. An example is general knowledge. This consists of several subdomains (sports, politics, history, cooking, films, etc.) that are correlated, but often not as strongly as items within these domains (Steger et al., 2019). Trying to make such a test unifactorial can result in ending up with questions that are very similar and only covering one particular knowledge domain, a phenomenon known as construct underrepresentation (Messick, 1989).

Of course, the situation is different if there is no clear elbow in the scree plot between factor 1 and factor 2 and if two or more factors are clearly above the random data. Then the test is measuring more than one factor. In some cases this makes theoretical sense (e.g. in personality tests where we expect 5 or 6 personality traits), in other cases the researcher will have to figure out what causes the unexpected extra factor(s). Also important if the test is multifactorial is to determine how much the factors correlate with each other. If there are high correlations between the factors, the test may still be measuring a single (hierarchical) construct. However, if the factors do not correlate (much) with each other, the test measures several traits (constructs). Then a sum score of the total test no longer makes sense (just as the sum of all the items of a personality test is meaningless) and separate scores must be calculated for the different factors. In that case, the reliability of the total test is also meaningless and the reliability for each factor must be determined separately. The test has become a collection of individual tests.

Most software packages work with Pearson correlation coefficients as the default. This is not always the best option. When the data are binary data (e.g., correct/wrong), tetrachoric correlations are better (Pearson, 1900). You get them in the psych() package by using the command:

fa.parallel(mydata, cor = “tet”).

For Likert scales, polychoric correlations are advised (Holgado–Tello et al., 2010), with the command:

fa.parallel(mydata, cor = “poly”).

Once you have decided on the optimal number of factors, you can get the solution for that factor analysis with the fa() function. The following command gives the outcome for a model with a single factor^5^:

fa(mydata, nfactors = 1).

If the data are noisy (reaction times, EEG signals), it may be good to ensure that the results are not too affected by a few outliers. One way to check the robustness of the solution is to use Spearman correlation instead of Pearson. This can be done with the command:

fa(mydata,nfactors = 1,cor = 'spearman').

An alternative control may be to use robust correlations. The *R* package WRS2 (Mair & Wilcox, 2020) can be used for this purpose. If the data are known to be inherently skewed (e.g. reaction times), it is good to check whether the solution is not more robust if logarithmic values are used instead of raw data. Importantly, the idea is not to try different analyses so you can present the best one (Head et al., 2015), but to see how analysis-dependent your conclusions are (Steegen et al., 2016). Ideally, you want a test with robust findings, where the outcome does not depend on finding the "right" analysis.

Further information about the use of factor analysis and how best to extract the number of factors can be found in Auerswald and Moshagen (2019), Sellbom and Tellegen (2019), or Goretzko et al. (2021).

### Item selection

Test development ideally starts with more items than needed, so that weak items can be pruned and the best items retained. An important criterion is the correlation between the item and factor performance. For a test measuring one factor, this can be gauged well by looking at the item-rest correlations. More in general, we can use factor loadings. This is how much an individual item loads on the factor.

Table 1 gives the outcome of such an analysis for the study with 24 items analyzed in Fig. 3. The data come from a vocabulary test, in which participants were given clues to 24 targets words and had to write down the word.^6^ In the table, we see that all items are doing well (high item-rest correlations and factor loadings), except for item 24, which was too difficult for the participants tested. Nearly all participants scored wrong on this item (mean accuracy = 3%).

**Table 1:** Item analysis of the 24 stimuli used in the study leading to the scree plot of Fig. 3

Item	Mean	SD	Item_rest	Factor_loading
item_1	0.92	0.27	0.50	0.78
item_2	0.93	0.26	0.60	0.89
item_3	0.91	0.29	0.53	0.81
item_4	0.94	0.24	0.58	0.92
item_5	0.87	0.34	0.57	0.80
item_6	0.77	0.42	0.43	0.62
item_7	0.92	0.28	0.62	0.92
item_8	0.88	0.33	0.60	0.85
item_9	0.57	0.50	0.40	0.58
item_10	0.83	0.38	0.55	0.74
item_11	0.77	0.43	0.46	0.61
item_12	0.66	0.48	0.47	0.65
item_13	0.49	0.50	0.42	0.61
item_14	0.72	0.45	0.43	0.59
item_15	0.62	0.49	0.55	0.73
item_16	0.85	0.36	0.58	0.80
item_17	0.74	0.44	0.47	0.63
item_18	0.66	0.47	0.52	0.68
item_19	0.78	0.41	0.59	0.79
item_20	0.34	0.48	0.35	0.53
item_21	0.71	0.45	0.57	0.75
item_22	0.71	0.45	0.52	0.69
item_23	0.49	0.50	0.48	0.64
*item_24*	*0.03*	*0.17*	*0.05*	*0.06*

All items are good (high item-rest correlations, high factor loadings), except for the last one

A second consideration to take into account when selecting items is to choose items with well-distributed levels of difficulty. Ideally, a test should contain items with an equal spread between easy and difficult. In Table 1, we see that items score between 94% correct and 34% correct (not including item 24). This is good, although there is some uninformative overlap in the difficulty levels of the items used and there is something to be said for a few more difficult items (with accuracies between 45 and 10%). Tests with a small range of item difficulty are more likely to result in ceiling effects for high-performing samples and bottom effects for low-performing samples.

**Fig. 4:**
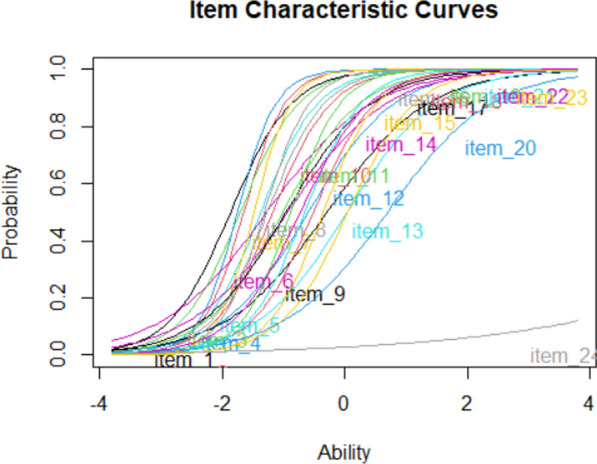
An IRT analysis shows how performance on items varies as a function of participant ability. Participants with low proficiency are expected not to know the item; participants with high proficiency are expected to know it. The left–right position of the item indicates the difficulty of the item (easy items are more to the left), the steepness of the curve item discrimination. Based on Rizopoulos (2007)

When accuracy data are used, item response theory (IRT) analysis is a nice addition (see the R file for code). It shows the expected performance of participants with different ability levels. Figure 4 shows the outcome of such an analysis for the test discussed in Fig. 3. Two elements are important. The first is item difficulty. This is represented by the left–right position of the items. The second element is the steepness of the curve. This is known as item discrimination.

**Fig. 5:**
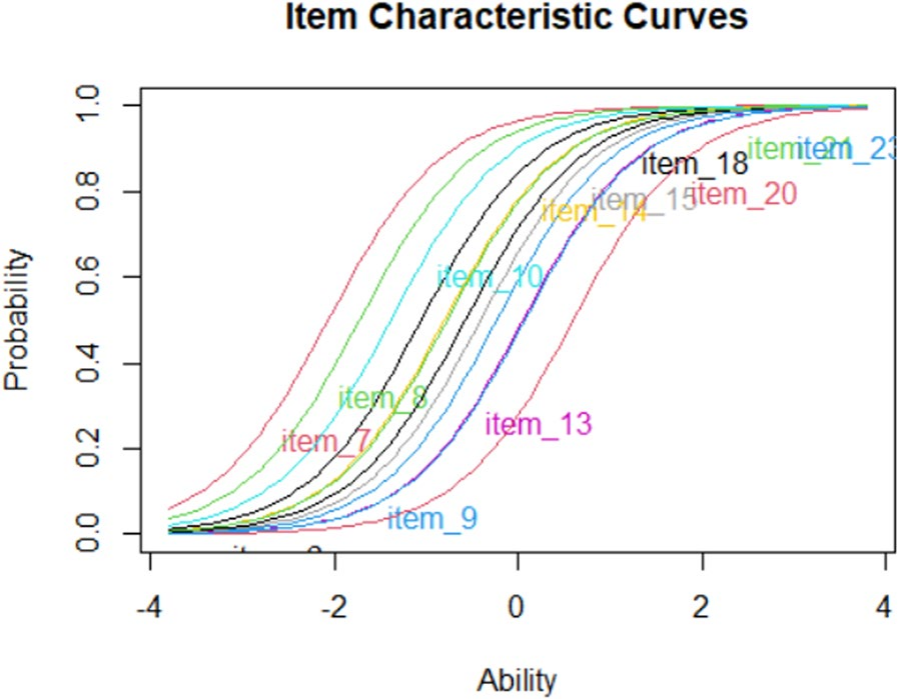
Profile of a test that approximates an ideal test. Item difficulties are equally distributed and have the same discrimination

In an ideal test, the items are well distributed in difficulty and all have high discrimination, as shown in Fig. 5. You can select the best items manually or with an algorithm (Kilmen & Bulut, 2023; Raborn & Leite, 2018).

### A second cross-validation study

At this point, the test developer designed stimuli, tested them on a large enough sample of participants, and pruned them to get a clear factor structure. Unfortunately, we are not there yet. A remaining danger is that the test developer selected items that will perform differently when retested. Items were chosen with a high correlation with the rest of the items or with a high factor loading, but as we saw in Fig. 2, estimates of correlations differ between studies. What the test developer still needs to know is whether the good items will hold up in a second, independent study. The probability of converging evidence is high if item selection and cross-validation are based on large samples, but not if they are based on small samples (Fig. 2). Therefore, a minimum sample size of 200 is recommended.

After running the new study, the data can be analyzed as in the first study. However, there are also more stringent techniques that can be used, such as confirmatory factor analysis (Schmitt, 2011) and assessment of measurement invariance (Luong & Flake, 2023; Vandenberg & Lance, 2000).

The data from the validation study can be used to create norms for the test. It is best to give the full distribution of the data so that test users have more information than just the mean and standard deviation. If norms are given, it is also necessary to be clear to which groups of participants they apply.

Hopefully, the independent validation study confirms the test's usefulness. However, any test developer will tell you that it often takes three, four, or even more rounds before a test is good. A lot depends on the quality of the materials you can start with. Developing good tests is much more demanding than many experimental psychologists realize.

### Summary test development

Table 2 summarizes the requirements for a good task of individual differences. Most have been covered in the previous text, but there are some new ones that may need some additional explanation.

**Table 2:** Criteria to use when developing a new test or evaluating a newly proposed test

*Minimum requirements*	
Good arguments are given for why a new test is needed Good arguments are given for the content of the test Analyses are based on representative samples of at least 200 participants Data loss is handled in a principled way The selection of items is well explained Reliability of each scale is at least .7 in an independent validation study The distribution of scores from the validation study is given The test is easily available Raw data and analysis code of all studies are freely available in a repository	
*Additional marks of distinction*	
Samples of 400 participants and more Test reliability greater than .8 Factor analysis and/or IRT analysis of the test Evidence of convergent validity or criterion validity Test–retest reliability in addition to internal consistency	
*Wrong criteria*	
A related test already exists The test must be validated on native English speakers	

A first extra criterion is the availability of the test. This may seem trivial, for why would someone develop a test and not make it available. However, a look at the (old) literature shows that such practice has been common, at least until the last decade. Authors described a test without giving access to it or telling readers how to obtain it. The idea seemed to be that readers should contact the authors, who then decide whether or not to give access to the test. Not only does this create a high barrier to the use of the test, but there is also much evidence that many authors no longer respond after the first few requests, either because they have other more pressing matters to attend to or because they have left academia and can no longer be reached. The unavailability of a test is a good reason not to publish the test in a scientific journal, because an article about a test is of little use to readers if they cannot apply it. Tests in cognitive research often use dedicated stimuli (e.g., images, videos, auditory stimuli), in which case the test standards apply only to the stimuli used. If new stimuli must be created, a new validation study is needed because researchers are in fact forced to create a parallel test.

Not only should the test be readily available, but also the data on which the test is based and the code used for the analysis. Test validation data are often large data sets that can be analyzed in different ways. This makes it important that users can check whether the test remains good if analyzed slightly differently. Transparency of analysis can do a lot here (Flake & Fried, 2020), but nothing trumps the availability of raw data. These also make it possible to test out new ways of analyzing and see how test performance changes as a function of them. Audits have shown that data without analysis code are difficult to understand and use correctly (Laurinavichyute et al., 2022). Therefore, it is important to provide the analysis code as well.

If a test meets all the criteria, and certainly the marks of distinction, the proposal is to publish the test. As explained in the introduction, any methodologically strong test is an asset to better capture the latent variables we are interested in. Related tests are not an annoyance but a strength, and very many tests are needed to systematically capture the variables we are interested in. Editors and reviewers often fear a deluge of tests and therefore want to limit them to those they find "really interesting." Elson et al. (2023) give some fuel to this fear, as they noted that there are more than 70 thousand tests available in APA PsycTests, most of which are barely used. But very few of the tests listed by Elson et al. (2023) meet the minimum requirements of Table 2, and even established tests need to be critically reviewed and updated regularly (Fried et al., 2022). Authors often have to create their own tests, not because they do not like using other people's tests, as Elson et al. (2023) claim, but because there are no good alternatives.

Guaranteed test publication when explicit criteria are met gives test developers guidance and assurance that their work will not be in vain. If desired, the criteria described here can be further refined (with specific statistical tests to run) so that evaluation of a new test is straightforward. Ideally, experimental psychology should have a journal where these tests are grouped so that they are easy to find.

Another criterion that is hard to defend is the bias of many editors and reviewers to reject tests not tested on English speakers. English-speaking researchers have the advantage that English is the lingua franca of science, giving Anglo-Saxon journals a big advantage. But if these journals want to promote international research, they cannot systematically refuse to publish articles from non-English-speaking parts of the world. Inclusion and diversity ring hollow if they stop as soon as an article is about a language you don't speak yourself. Editors can reduce this bias by seeking reviewers who know the language of the test.

Sometimes researchers underestimate how strong the effects of publication bias are. One should not be surprised at the low quality of our tools, if we deny rewards to those who develop them. Not being able to publish tests in international journals kills the careers of researchers who have the courage to work on them and who often have to leave academia early because there is no more money for what they are doing. This is especially true in non-English-speaking countries, if publications in international (English-language) journals are required for career advancement. We cannot have it both ways: If we want good tests of individual differences in cognitive research, we must reward authors who create them. We must not nip in the bud as many new tests as possible, but give them a chance to compete with existing ones, as long as they meet quality criteria. In return, test developers must provide easy and affordable (ideally free) access to the materials for which they want a publication, so that researchers can build on the tasks.^7^

## Evaluating and strengthening existing tests

Just as important as developing new tests, is continually evaluating and improving existing ones. This touches on Kane's (2013) target level: How confident can we be that a test is a good measure of the construct it is intended to measure? Kane called it the appraisal of a test and he attributed particular relevance to appraisal by neutral, skeptical evaluators. Hopefully, the replication crisis of the 2010s in psychology has convinced us all of the importance of independent replication studies (Nosek et al., 2022; Zwaan et al., 2018), also in test construction (Lilienfeld & Strother, 2020).

Ideally, the researchers who developed the test already conducted a preliminary evaluation of its validity. This typically involves administering the test along with other related measures or criteria to establish that the test is significantly correlated with the related constructs and not significantly correlated with unrelated constructs.

Continued evaluation is necessary, however, because the true value of a test only becomes apparent when the test is used in new samples that are independent of the sample used for test construction. As we saw above, this is why we need a second, independent validation sample in test construction (at that point, it is easy to measure a few additional related and unrelated tests or criteria). Usually, however, that sample is collected by the same group of researchers as the one who developed the test. Therefore, further research by neutral users is important. If a test is used in multiple studies, its pattern of intercorrelations becomes clear and item analysis may reveal poor performance of a few items across studies.

Another way to improve a test is to see if changes in method increase the criterion validity of a test. This assumes, of course, that one has a validation criterion. For example, Zhang and Zhang (2022) examined which vocabulary test is the best predictor of language comprehension. Different types of vocabulary tests can be made: spoken vs. written language, yes/no questions, different types of multiple choice questions, whether participants can select the answer (recognition) or have to produce it (recall), and so on. Zhang and Zhang (2022) conducted a meta-analysis to find out whether all of these formats are equally good, or whether some are better than others. They found that meaning recall (giving the meaning of target words) was the best predictor of reading comprehension, while form recall (giving the target word for a described meaning) was the best predictor of listening comprehension.

Evaluation of tests also examines the extent to which tests claiming to measure the same skill converge with each other. This is known as the jingle fallacy (Gonzalez et al., 2021; Kelly, 1927): the fact that a common label makes us expect two tests to measure the same construct, when this need not be the case. Interesting examples come from screening instruments for mental disorders. For example, Fried et al. (2022) examined seven commonly used scales for depression and found that they contained 52 different symptoms, 40% of which appeared in only one scale. While such diversity need not be a bad thing for research, it is important to know how well the different scales converge, especially if they are used for clinical diagnosis and have real-life implications. Similarly, we may wonder to what extent different measures of working memory or executive function converge on the traits they claim to measure (Miyake et al., 2000; Muffato et al., 2023; Rey-Mermet et al., 2018; Snyder et al., 2021).

Equally important is making sure that a test measures the function it purports to measure and not some related function. This is known as the jangle fallacy (Gonzalez et al., 2021; Kelly, 1927; Larsen & Bong, 2016): the fact that using different labels causes us to expect two tests to measure different things, when in reality they measure the same thing. For example, Draheim et al. (2022) noted that some attention studies examine inhibition while others examine attention control, and it is not clear whether these two labels are different or not (see also Necka et al., 2018, and Strand et al., 2020, for further examples of jingle-jangle fallacies in cognitive research).

Wulf and Mata (2023) showcased the potential of artificial intelligence and large language models to detect jingle and jangle fallacies in the construction of personality scales. Their findings raise the prospect of using deep learning networks to identify patterns of interrelationships among experimental tasks, further improving the validity of experimental tasks for research on individual differences.

When evaluating tests or tasks against established theories, researchers will rapidly encounter the limitations of exploratory factor analysis. A more suitable approach in this context is confirmatory factor analysis (Jackson et al., 2009; McNeish & Wolf, 2023; Schmitt, 2011; Schreiber et al., 2006). However, confirmatory factor analysis has its own limitations, as complex models often deviate significantly from the theoretical template (or the findings of previous studies), making it challenging for researchers to draw clear conclusions. As an alternative that addresses this issue, structural equation modeling (SEM; see below) proves to be a valuable tool. SEM allows researchers to start with a theoretical model and then make adjustments based on the new data collected. This iterative approach enables a more fine-tuned alignment between the theoretical model and the empirical evidence (Cheung et al., 2023; Marsh et al., 2014, 2020).

When evaluating tests, it is tempting to focus on a single, best test and recommend it for future use. An alternative within the multitrait multimethod approach is to look at how much different tests contribute to a latent variable and how much this latent variable correlates with other latent variables. This brings us to Kane's (2013) construct level.

### Combining tests to gain better understanding

According to the structural equation approach, performance on a test is the outcome of three sources of variance: (1) the skills measured by the test, (2) the method used for the test, and (3) measurement error. The last part is estimated by looking at the reliability of the test (a test with high reliability has less measurement error than a test with low reliability). One way to discriminate between the first two parts is to take more than one test for the skills you want to measure.^8^

An example can be found in Vermeiren et al. (2023, study 5). They examined the correlation between vocabulary knowledge and reading comprehension. Instead of using one test for each skill, they used four tests of reading comprehension from different sources and three vocabulary tests.

Table 3 shows the correlations between the tests and also the reliability of each test (on the diagonal). The correlations between the comprehension tests averaged 0.46; those between the vocabulary tests were higher, averaging 0.74 (these tests also had higher reliability). The correlations between vocabulary tests and comprehension tests averaged 0.41.

**Table 3:** Correlations between four reading comprehension tests (Comp 1–4) and three vocabulary tests (Voc 1–3)

	Comp1	Comp2	Comp3	Comp4	Voc1	Voc2	Voc3
Comp1	***.48***						
Comp2	.51	***.76***					
Comp3	.40	.40	***.88***				
Comp4	.46	.60	.42	***.74***			
Voc1	.34	.23	.43	.37	***.89***		
Voc2	.38	.30	.51	.41	.81	***.91***	
Voc3	.46	.44	.54	.52	.68	.72	***.93***

The numbers on the diagonal (in bold and italics) give the reliability of each test

On the diagonal the reliability of each test (omega total). Number of participants tested = 182

We can run an exploratory factor analysis to see how many factors are needed to account for the pattern of intercorrelations in Table 3, how much the factors correlate with each other, and how much the tests load on each factor. An alternative is an exploratory graph analysis (Christensen & Golino, 2021; for examples of use in experimental psychology, see Goring et al., 2021; Hintz et al., 2024). This provides a network of the tests and clusters them on the basis of statistical criteria. For this we can use the following code in R:

library(EGAnet).

EGA1 <—EGA(Vermeiren_study5).

**Fig. 6:**
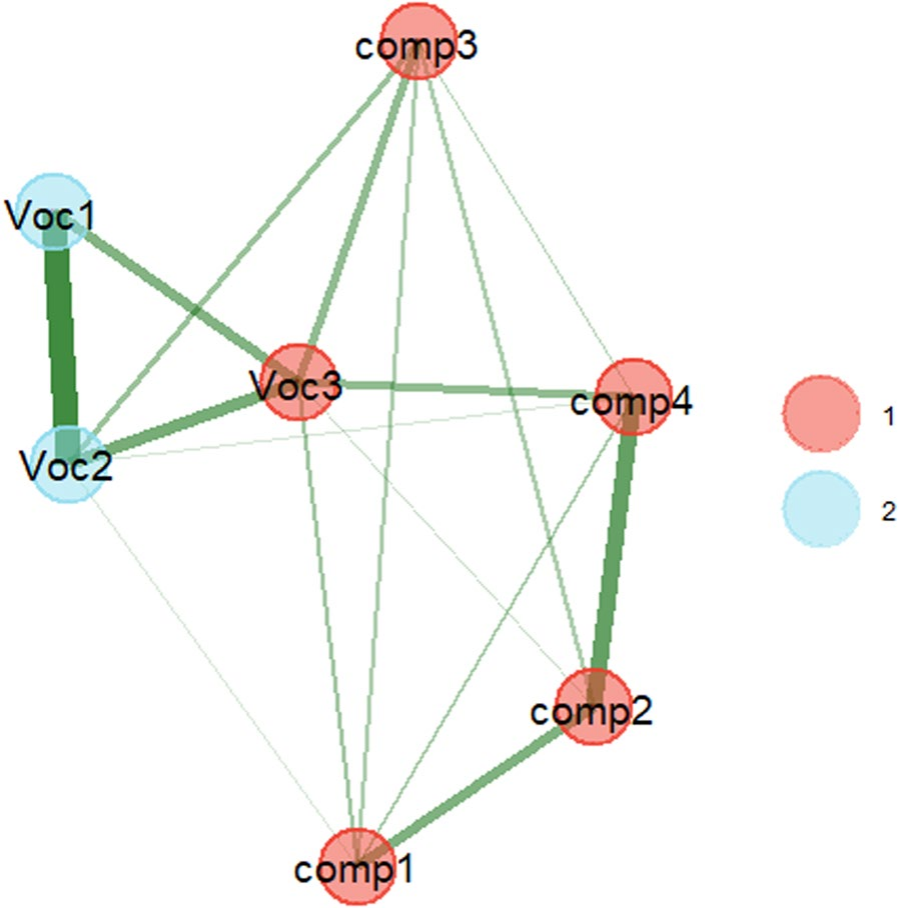
EGA outcome of the data shown in Table 3. It suggests that the vocabulary test Voc3 belongs to the cluster of reading comprehension tests rather than the cluster of vocabulary tests. It also shows strong correlations between Comp3 and two of the vocabulary tests

Figure 6 shows the result. It suggests that the vocabulary test Voc3 belongs to the cluster of reading comprehension tests rather than the cluster of vocabulary tests. It also shows that there are strong correlations between Comp3 and two of the vocabulary tests. The ambiguous positions of Voc3 and Comp3 are also apparent when bootstrapping is used to check solution stability (Christensen & Golino, 2021). In almost half of the solutions, they end up in one cluster or in the other. This suggests that they are not pure measures of vocabulary or reading comprehension.

The ambiguous nature of Voc3 is interesting because this vocabulary test comes from the Nelson-Denny test (Brown et al., 1993), which is best known for its test of reading comprehension. It is not inconceivable that the authors of the Nelson-Denny test selected vocabulary items that not only did well according to traditional item selection procedures, but also correlated well with reading comprehension. Similarly, Comp3 differed from the other tests of reading comprehension because it was the only test with time constraints: Participants not only had to read carefully, they also had to read quickly.

A technique that allows tests to load on different latent variables is structural equation modelling (SEM). A much-used R package is lavaan (Rosseel, 2012). The following code is what we need (see https://lavaan.ugent.be/tutorial/ for more information):

library("lavaan").

model <—'.

# measurement model.

comprehension =  ~ comp1 + comp2 + comp3 + comp4 + Voc3.

vocabulary =  ~ Voc1 + Voc2 + Voc3 + comp3.

'

Fit <—sem(model, data = Vermeiren_study5).

summary(fit, fit.measures = TRUE, standardized = TRUE).

**Fig. 7:**
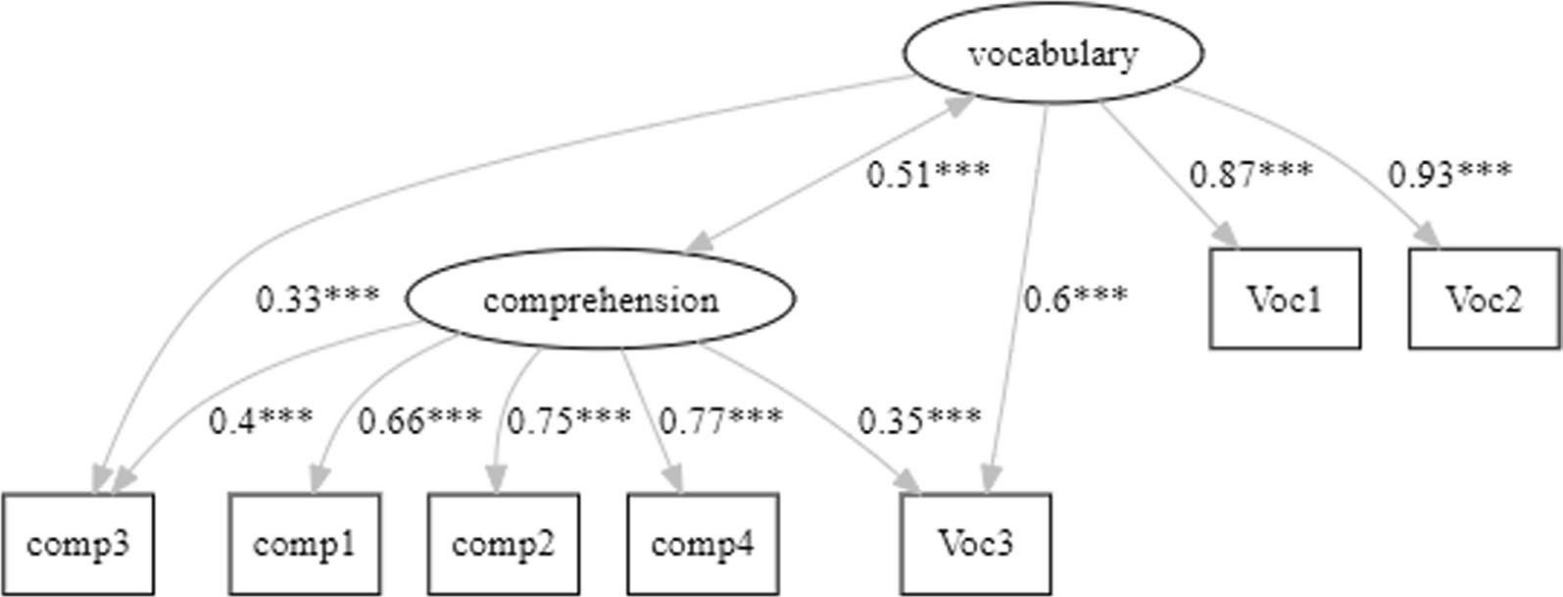
Structural equation model of the data shown in Table 3. It suggests that the correlation between vocabulary knowledge and reading comprehension increases if both skills are estimated on the basis of multiple tests (based on lavaanPlot; Lishinski, 2018)

Figure 7 shows the graphical outcome of the SEM analysis (Lishinski, 2018). Fit measures indicate that the model is a good description of the pattern of intercorrelations (CFI = 0.995, TLI = 0.991, RMSEA = 0.038, SRMR = 0.036).^9^

The SEM analysis also suggests that the correlation between the two constructs is 0.51. This is higher than the correlation of 0.41 suggested by the average correlation between the individual tests, consistent with the observations that test include measurement error and that no test on its own is an ideal measure of a latent variable. SEM allows us to examine not only how constructs relate to each other, but also to what extent tasks are good measures of the constructs.

Völker (2020) provides another example of how combining tests can provide a better picture of the relationship between constructs (and ultimately their construct validity). A recurring question in psychological research is to what extent emotion understanding depends on intelligence. Is understanding emotions a separate skill or a jangled concept that is part of general intelligence (e.g., MacCann et al., 2014)? Many of the skills involved are based on experimental tasks. For example, emotion recognition is studied by looking at how well people can distinguish emotions in pictures of the eyes (Baron-Cohen et al., 2001; Franca et al., 2023) or in short video clips (Schlegel & Scherer, 2016).

Olderbak et al. (2019) conducted a meta-analysis of studies on the relationship between emotion processing and intelligence. They distinguished between four aspects of emotion processing: emotion recognition, facilitating thoughts using emotion, managing emotions, and understanding emotions. For the first three aspects, the correlation with intelligence was around *r* = 0.2; for emotion understanding, it was higher (*r* = 0.45). There was no major difference between tests measuring fluid intelligence (reasoning efficiency) and tests measuring crystallized intelligence (mainly retrieving verbal information from memory). At first glance, these findings are reassuring because they suggest that emotion processing is largely distinct from intelligence. Only for emotion understanding is there a fairly high correlation, but this can be understood given that tests of emotion comprehension tend to be verbal tests (requiring participants to distinguish between emotion labels and situation descriptions).

Völker (2020) presented all four emotion tasks to a group of students along with three tests that gauged fluid intelligence (numerical, figural and verbal reasoning) and three tests that gauged crystallized intelligence (general knowledge, verbal fluency and word meaning). Table 4 shows the correlations between the tests. They are largely consistent with the estimate of *r* = 0.2 from the meta-analysis of Olderbak et al. (2019), except for emotion understanding, which seemed to have correlations in line with the other emotion processes.^10^

**Table 4:** Correlations between performance tasks of emotion processing and intelligence, reported by Völker (2020)

	2	3	4	5	6	7	8	9	10
1. Emotion recognition	.25	.07	.26	.11	.20	.16	.26	.15	.20
2. Emotion understanding		.10	.07	.22	.16	.11	.27	.04	.15
3. Emotion regulation			.19	.06	.07	.10	.06	-.15	-.01
4. Emotion management				.09	.13	.08	.23	.22	.05
5. Numerical reasoning					.46	.22	.27	.21	.28
6. Figural reasoning						.39	.43	.30	.21
7. Verbal reasoning							.24	.16	.20
8. General knowledge								.24	.37
9. Verbal fluency									.16

Number of participants = 178. No test reliabilities were reported

**Fig. 8:**
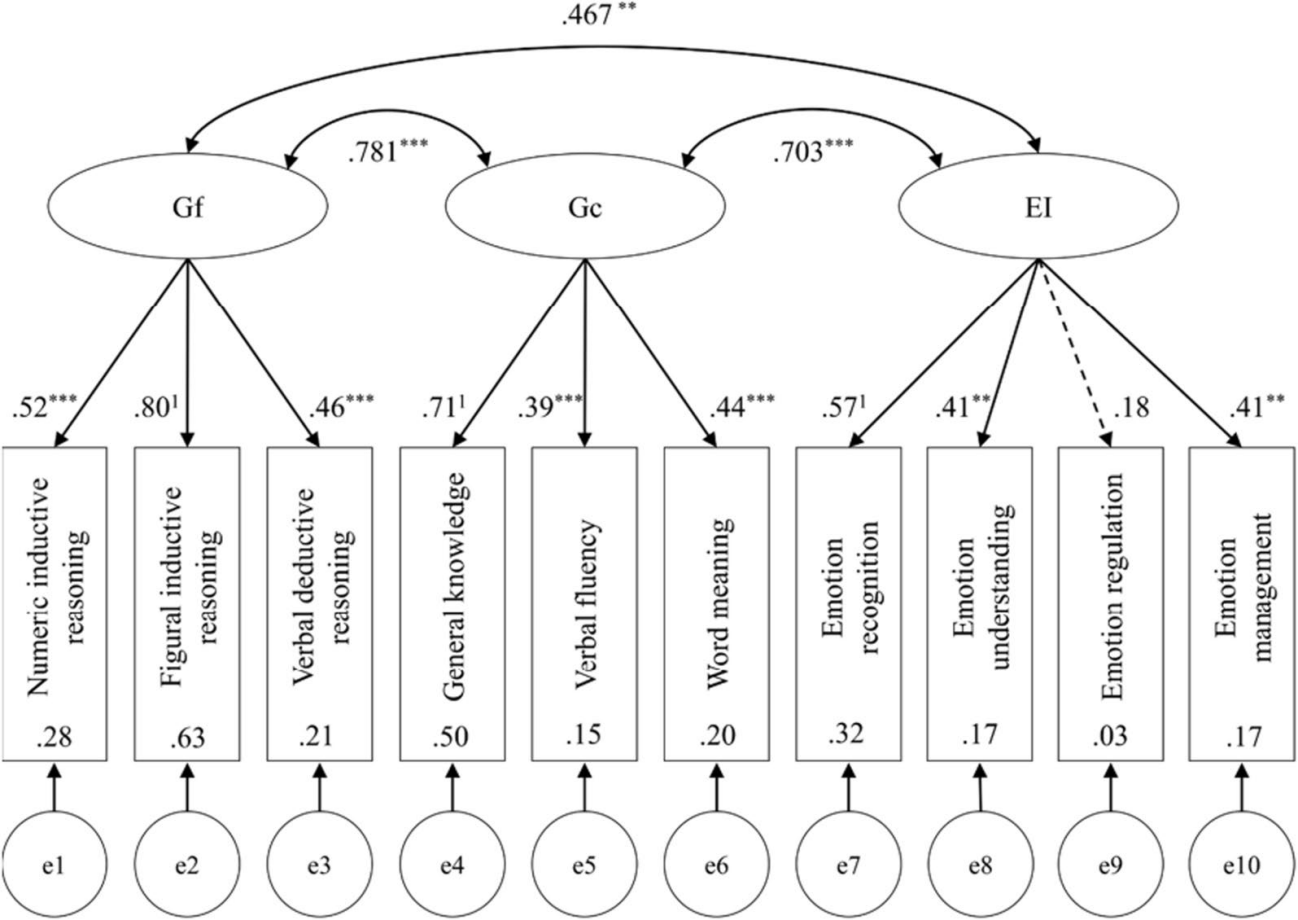
SEM analysis of the data in Table 4. Figure copied from Völker (2020; Fig. 2). *Gf* Fluid intelligence, *Gc* Crystallized intelligence, *EI* Emotional intelligence

SEM gave a very different picture, as shown in Fig. 8. Now the estimated correlation between emotional intelligence (EI) and fluid intelligence (Gf) was 0.47. The estimated correlation with crystallized intelligence (Gc) was even 0.70. One caveat to these findings is that all of the original correlations were low, suggesting that the reliability of the individual tasks was low. This means that there was quite a bit of extrapolation about theoretically expected values for reliable tests. It is much better to aim for tests with good reliability so that not much extrapolation is needed. This increases the likelihood that the findings will be robust.

Schmiedek et al. (2014) reported a similar finding in working memory research. They started from a meta-analysis suggesting a correlation of only *r* = 0.2 between complex span tasks and n-back tasks, two key measures of working memory capacity. This seemed to suggest that the two types of tasks cannot be used interchangeably as working memory measures. However, using three tasks for each latent variable, Schmiedek et al. (2017) observed a correlation of *r* = 0.69 between the latent variable estimating complex span performance and the latent variable estimating n-back performance. Both latent variables correlated strongly with a latent variable of working memory and the latter in turn explained more than 71% of the variance in a latent variable of reasoning measured by three different reasoning tasks. Combining different types of tasks was better than using tasks of a single type, suggesting that highly correlated indicators provide poorer coverage of the construct space. So rather than focusing research on finding a single task that best captures the construct under study, experimental psychologists are advised to look for combinations of tasks that provide the most information (see also Marcusson-Clavertz et al., 2022).

The possibility that low correlations between tests do not necessarily imply a lack of relationship between them poses a challenge in establishing a minimum criterion for validity correlations. Although a statistically significant correlation seems a necessary condition, the magnitude of a significant correlation can be low with a large sample size. Tentatively, a minimum correlation of 0.2 could be advanced as the smallest correlation coefficient of interest (SCCI), based on the observations that this is the typical correlation found between related variables in psychology (Bosco et al., 2015; Stanley et al., 2018) and that unrelated variables rarely show a correlation of 0.0 (Orben & Lakens, 2020). Hopefully, the accumulation of evidence from multiple studies will lead to subject-specific validity thresholds. Based on the findings in Tables 3 and 4, we would consider a correlation of 0.2 between a vocabulary test and a reading comprehension test to be low, whereas it seems to be more common in working memory research. A better understanding of these problems will only come if researchers are rewarded for doing large-scale studies using multiple tests with high reliability and validity.

A reviewer expressed concern that administering multiple tasks per trait may create time constraints and introduce possible interference effects in a typical experiment. While acknowledging these issues, it is crucial not to let practical limitations dictate the quality of a study. The primary question should not be “How much can we investigate in one hour?” but “How much time is necessary to adequately assess the constructs?”, similar to prioritizing the question "How many participants are required to get stable correlation coefficients".

## Conclusion

Researchers in experimental psychology are increasingly looking at individual differences to test theories of cognitive functioning. In doing so, they face two challenges: (1) they need knowledge that is often not taught to them, and (2) they must convince editors and reviewers who do not have the required knowledge. This tutorial is an attempt to improve the situation.

Lilienfeld and Strother (2020) listed four myths about psychological measurement among non-experts (experimental psychologists):

1. We can safely rely on the name of a measure to infer its content and validity (this myth ignores the many jingle and jangle fallacies already discovered).
2. Reliability is not a major concern for laboratory measures (this myth ignores the fact that research on individual differences is not possible with variables that lack reliability).
3. Using measures that are difficult to collect obviates the need for large sample sizes (this myth ignores the fact that statistics is blind to the cost of collecting data).
4. Convergent validity data afford sufficient evidence for construct validity (this myth ignores the possibility that a full set of related tests can measure something different than imagined).

To these, the present article adds seven more myths:

5. If we already have a test, we don't need a new one. A new test is only interesting if it is much better than the existing test or if it measures a new function (this myth ignores the distinction between test and skill and confuses manifest variable with latent variable).
6. Established tests need no scrutiny (this myth ignores the possibility that the original test was not as good as claimed and that historical changes may reduce the usefulness of the test).
7. Laboratory studies need not use established tests and protocols because there is no need to compare performance between studies (this myth ignores the importance of cumulative science and ignores the importance of establishing the validity of the task beyond content validity).
8. A newly developed test cannot be used in research as long as there are questions about construct validity (this myth ignores the fact that construct validity is multifaceted and requires more scrutiny for tasks with social relevance than for tasks used in basic research to understand cognitive processes).
9. Linear (mixed effects) regression is all that is needed in experimental research (this myth ignores the contributions of item analysis,^11^ factor analysis, and structural equation modeling).
10. All information in laboratory studies can be obtained from a single study (this myth ignores the importance of cross-validation in independent studies).
11. Low correlations between tasks indicate that the tasks measure unrelated skills (this myth ignores the possibility that low correlations between tests can be due to measurement error and to the fact that tests only measure the underlying skill to some extent).

Overcoming these misconceptions will ensure that research on individual differences contributes more fully to understanding the cognitive processes underlying human performance.

## Data Availability

All data and stimulus materials are available at anonymized.

## Abbreviations

FA: Factor analysis
IRT: Item response theory
SD: Standard deviation
SEM: Structural equation modelling

## Footnotes

1 Participants have to name the color of words. A distinction is made between congruent trials in which the color of word is the same the meaning of the word (RED printed in red) and incongruent trials in which the color of the word differs from the meaning of the word (BLUE printed in red).

2 Even a parallel test can be interesting. This is a test developed along exactly the same lines as an existing test (e.g., a vocabulary test with new words). Such a test may be of interest if participants need to be tested repeatedly and if there are concerns that using the exact same test twice may affect performance.

3 Code for all R analyses reported in this ms can be found at https://osf.io/9hv5j/. Some analyses can also be done in the free package jamovi (The jamovi project, 2022), which is user-friendlier but limited in the number of analyses it can do and in the size of the datasets it can handle.

4 An alternative to factor analysis is principal component analysis. The latter will not be discussed here. See Sakaluk and Short (2017) for a tutorial.

5 Or fa(mydata,nfactors = 1,cor = 'tet').

6 The author thanks Amber Callens for collecting the data.

7 University management often pushes to commercialize tests. This creates a huge barrier to using the test, which usually results in the test being rarely used (purchased) and a loss for everyone involved.

8 Having more than one test per skill has the added advantage that a test with low reliability does not ruin an entire part of the analysis. If you have only one test for a skill, then low reliability of the test (e.g., due to range restriction) is impossible to overcome, even if you have used a well-established test. Then it is impossible to know whether the skill is unrelated to other skills or whether the test was too poor to show the relationship.

9 CFI and TLI indicate goodness of fit and are ideally above .95; RMSEA and SRMR are error rates and are ideally below .05.

10 Keep in mind that the meta-analysis of Olderbak et al. (2019) is based on data such as those reported by Völker (2020).

11 Unless the person making the claim knows that mixed effects modeling can be used for item analysis (De Boeck & Partchev, 2012).
